# Ancestry deconvolution and partial polygenic score can improve susceptibility predictions in recently admixed individuals

**DOI:** 10.1038/s41467-020-15464-w

**Published:** 2020-04-02

**Authors:** Davide Marnetto, Katri Pärna, Kristi Läll, Ludovica Molinaro, Francesco Montinaro, Toomas Haller, Mait Metspalu, Reedik Mägi, Krista Fischer, Luca Pagani

**Affiliations:** 1 Institute of Genomics, University of Tartu, Riia 23b, 51010 Tartu, Estonia; 2 Institute of Molecular and Cell Biology, University of Tartu, Riia 23, 51010 Tartu, Estonia; 3 Department of Epidemiology, University Medical Center Groningen, Hanzeplein 1, 9713 GZ Groningen, The Netherlands; 4 Institute of Mathematics and Statistics, University of Tartu, Narva mnt 18, 51009 Tartu, Estonia; 5 Department of Biology, University of Padova, Via Ugo Bassi 58/B, 35131 Padova, Italy

**Keywords:** Predictive medicine, Genetic association study, Genetic variation

## Abstract

Polygenic Scores (PSs) describe the genetic component of an individual’s quantitative phenotype or their susceptibility to diseases with a genetic basis. Currently, PSs rely on population-dependent contributions of many associated alleles, with limited applicability to understudied populations and recently admixed individuals. Here we introduce a combination of local ancestry deconvolution and partial PS computation to account for the population-specific nature of the association signals in individuals with admixed ancestry. We demonstrate partial PS to be a proxy for the total PS and that a portion of the genome is enough to improve susceptibility predictions for the traits we test. By combining partial PSs from different populations, we are able to improve trait predictability in admixed individuals with some European ancestry. These results may extend the applicability of PSs to subjects with a complex history of admixture, where current methods cannot be applied.

## Abstract (web summary)

Polygenic scores are believed to hold future promise for trait prediction and personalized medicine, but are sensitive to demographic history. Here, Marnetto *et al*. develop partial polygenic scores supplemented with local ancestry deconvolution which improves prediction accuracy into recently admixed European populations.

## Introduction

Polygenic Scores (PSs) are computed by summing the contribution of many associated alleles across the genome^1,2^. These contributions are weighted by allele effect sizes. Such effect sizes are extrapolated from Genome Wide Association Studies (GWAS)^3,4^ carried out in a specific population, or across multi-ethnic sample sets^5–7^. Most of the times, associated SNPs are thus merely correlated with a phenotype and are not really causal^3^. Furthermore, as environment interactions, Linkage Disequilibrium patterns, allele frequencies and rare polymorphisms are often population specific, effect sizes might be in part population dependent^3,8,9^. This may lead PS to exhibit a directional bias and a lower predictivity in individuals from populations not closely related to the one where the GWAS study was performed^10–14^ or even from the same population as it was shown for UK and Finnish cohorts^15–17^. This is particularly problematic with recently admixed individuals, where the various ancestries composing a given genome may be closely or distantly related to the population used to infer the adopted genetic effect sizes^18^. Admixed individuals are indeed expected to constitute a considerable portion of contemporary and future societies^19^. Therefore, it is crucial to include them within the promising vault of the emerging predictive and personalized healthcare.

While any human genome can be seen as the mixture of its ancestors, here we focus on individuals such as African-Americans in the USA; Afro Caribbeans in the UK; Central and South Americans with European descent; Ethiopians and North Africans and others, who trace their ancestry from a recent admixture event (less than 100 generations ago)^20^ between two or more human populations separated by at least 1000 generations of independent genetic drift. Over these few generations, recombination events created a unique tiling of ancestry blocks for each recently admixed individual^21,22^. Importantly, even if the current PS models assume so, there is no evidence on whether any expressed phenotype of an admixed genome should be seen as the linear sum of ancestry-specific effect sizes calculated along the genome, considering its specific local ancestry composition. In addition, the genetic effect sizes reported for certain ancestries may be more accurate than others, due to the greater amount of studies available^10,23^. Thus, local PS accuracy may vary along the genome and among individuals, depending on the relative fraction and on the particular tiling of a given ancestry each person has.

PS transferability has been proved exceptionally difficult across deeply divergent populations^10–14,18,23^, while ancestry deconvolution softwares display reliable performances in discerning genetic contributions from these groups of individuals^22,24^. Although human genetic diversity is distributed as a continuum across all continents, here we focus on the extremes of such a genetic gradient (i.e. genetic drift components that are modal in European, East Asian or Sub-Saharan African populations) to ease a preliminary exploration of the viability of our approach, and assume within population stratification as a lesser, yet important, source of bias. Hence, the drift components, or genetic ancestry labels, used here should just be seen as proxies for a broader gradient, not necessarily overlapping with cultural identity or self reported ethnicity of the analyzed individuals. Accordingly, we relied on a sample set including 120 Ethiopians, 100 Egyptians^25^ and 49 African-Americans^26^, all of which resulted from the admixture of African and West Eurasian populations approximately 100, 30, and 6 generations ago, respectively^20,25^. We also included 22054 samples from UK Biobank^27^, 5793 of which present an admixed genetic background(Supplementary Table 2). These samples display a polarized combination of highly studied and severely understudied genomic segments.

We focus on PS of four thoroughly studied traits (Type 2 Diabetes (T2D)^28^, height^29^, Body Mass Index (BMI)^29^ and breast cancer^30^. By introducing the concept of partial PS and applying it on the sample groups above, we find that a small portion of the genome is enough to improve trait predictions and that such approach can be used to correct for population level PS bias. Finally, we test the predictivity of ancestry specific partial PSs and their combination on datasets for which both phenotypic and genotypic information are available, namely the Estonian Biobank^31^ and the UK Biobank^27^. The results show that, when GWAS data are available for more than one ancestry, the combination of multiple partial PSs improves trait predictability in individuals with a mixed genetic background.

## Results

### Proposed model and workflow

As introduced above, current PSs are often poorly transferable across populations. Considering PS as a normally distributed variable which is partially correlated with the trait of interest, we can mimic the poor transferability of PS across populations by applying a directional bias to the PS and reducing the correlation with the trait. In particular, this decrease in correlation and therefore predictivity can be absent, with fully transferable PSs, or complete, leading to a PS altogether disconnected with the trait of interest.

Let us consider an admixed genome which descends with proportion *p* from population *A* and with proportion 1−*p* from population *B*. We can compute a PS for this individual adopting summary statistics obtained from a GWAS performed in population *A*, PS_A_. We define a model to simulate such admixed genome by combining an unbiased PS with a biased and poorly predictive PS with a proportion *p* in one single individual. As expected in case of poor PS_A_ transferability, the proportion of ancestry *A* (*p*) is positively correlated with predictability of PS_A_ for the trait of interest (Supplementary Fig. 1, yellow line).

We can also define ancestry specific partial PS (aspPS_A_) as a proxy for the total standardized PS that uses only the genomic portion pertaining to the ancestry *A*, losing therefore information as *p* decreases, but remaining virtually unbiased. A model including this metric (Supplementary Fig. 1, blue line) would outperform the traditional PS (PS_A_) in case of high directional bias and *p* and, with low transferability, even a model that takes into account *p* as a separate variable to explain the directional bias (Supplementary Fig. 1, red line). The limitation of not considering parts of the genome, makes the aspPS only partially useful in improving trait predictability. On the other hand, considering the availability of another PS (PS_B_) originating from a GWAS on a population closer to the other ancestry *B*, and therefore not suffering of transferability issues, one can imagine to combine the two aspPS, weighting for *p*, in order to obtain a combined ancestry specific PS (casPS). In our simulation, the casPS would outperform the traditional PS in all cases where PS transferability represents a problem and where a sufficiently predictive PS is present for the two ancestries independently (Supplementary Fig. 1, purple line).

**Fig. 1:**
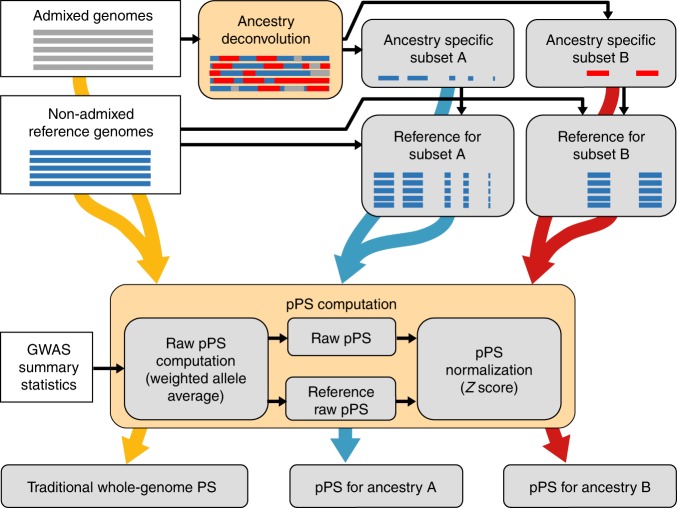
Schematic workflow. A graphical representation of the workflow we adopted to obtain normalized PS and ancestry specific pPS. White boxes represent input data, the two key steps of ancestry deconvolution and partial PS computation have an orange background.

We thus proceeded defining a way to compute partial PS (pPS), a statistic that estimates the total standardized PS using only a subset of the genome, as

1 $${{\rm{pPS}}}_{j}=\frac{{\overline{x}}_{j}^{\prime}-{\mu }_{\overline{x}^{\prime} }}{{\sigma }_{\overline{x}^{\prime} }},$$

where ${\overline{x}}_{j}^{\prime}$ is the raw partial PS for individual *j*, defined as the weighted average of associated allelic states using only a fraction *p* of all available SNPs. In turn, ${\mu }_{\overline{x}^{\prime} }$ and ${\sigma }_{\overline{x}^{\prime} }$ are, respectively, mean and standard deviation of the raw PS ($\overline{x}^{\prime}$) in a population of reference. Hence, pPS is essentially a Z-score which yields the traditional standardized PS when applied to the full genome. Furthermore, we call the pPS calculated on ancestry specific portions of a given genome “ancestry-specific pPS” (aspPS). The model described above ignores entirely the problem of coupling the correct portions of the genome with the appropriate PS, especially important due to the unique tiling of ancestry blocks and with unequal genomic patterns of phenotypic association. We overcome this obstacle by defining the genomic subsets used to compute aspPS through an accurate local ancestry deconvolution process (Fig. 1).

### Local ancestry deconvolution

We started by assessing the accuracy of ELAI^24^, a local ancestry inference (LAI) or  deconvolution (LAD) software, in simulated scenarios resembling our studied populations. We tested several combinations of admixtures between deeply divergent populations (i.e that trace their ancestry to East Asia, Africa and Europe) and number of generations elapsed since the admixture (Supplementary Fig. 2), reporting in all cases a classified accuracy equal or higher than 0.98 when using 0.9 as the minimum inferred ancestry dosage. We then subdivided the real admixed whole genome sequences of individuals with both African and West-Eurasian background into their ancestral components through this process. This gave us two genomic portions, one African- and one European-related, together with an unassigned fraction for each genome (Supplementary Fig. 3).

### Population-level aspPS distributions

**Fig. 2:**
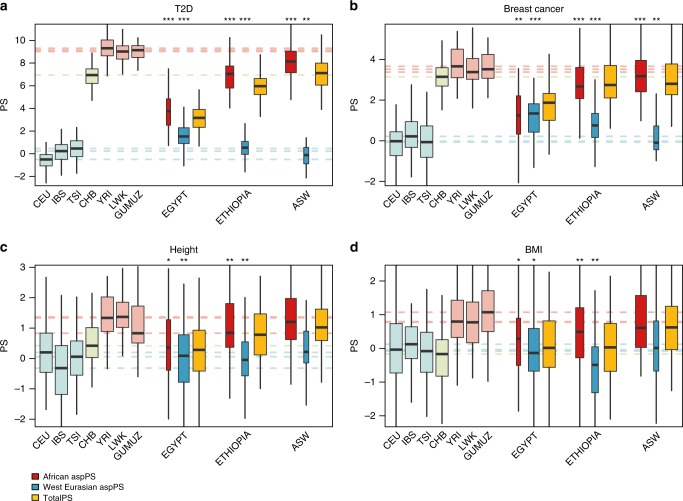
Population-wide Polygenic Scores (PS) and ancestry specific partial PS. PS distributions for seven reference populations (pastel colors), three admixed populations (yellow) and their relative ancestry specific partial PS (red and blue). Reference population medians are represented with dashed lines. The width of the boxplots is proportional to the median size of the ancestry fraction used to compute each aspPS. Four different PS for different phenotypes are shown: (**a**) T2D^28^, (**b**) breast cancer^30^ (**c**) height^29^, (**d**) BMI^29^. Significant differences with randomly assigned ancestral components are encoded as: *: *p* ≤ 0.05, **: *p* ≤ 0.005, ***: *p* ≤ 10^−5^, (one-sided Wilcoxon signed-rank test). Sample sizes and exact *P*-values are reported in Supplementary Data 1. For each distribution, the box represent the interquartile range (IQR = *Q*_3_−*Q*_1_), the line across the box indicate the median, the whiskers extend to the most extreme data points within *Q*_1_−1.5IQR and *Q*_3_ + 1.5IQR, outliers are omitted. CEU: North-West Europeans from Utah; IBS: Iberians from Spain; TSI: Tuscans from Italy; CHB: Han from Beijing; YRI: Yoruba from Nigeria; LWK: Luhya from Kenya; GUMUZ: Gumuz from Ethiopia; EGYPT: Egyptians; ETHIOPIA: Amhara, Oromo, Wolayta and Ethiopian Somali from Ethiopia; ASW: African-Americans from South-West USA.

The PS population distributions of the four traits considered here, calculated on the whole genomes of our reference panel and standardized on the European populations (CEU, IBS, TSI, light blue boxes in Fig. 2) show that the effect sizes ascertained on European populations may generate spuriously positively- or negatively-shifted PS distributions in non-European groups (i.e. YRI, LWK, CHB...), as observed before^12^. As mentioned in the introduction, this phenomenon is combined with poor predictive performance of PS based on European effect sizes applied to those non-European populations^18^. The same pattern is observed in Egyptians, Ethiopians, and African-Americans, most remarkably for T2D (Fig. 2a), where the PS distribution can be described as a linear combination between a 0-centered European and a positively-shifted African distributions. When computing aspPS instead, the two ancestries always returned distributions shifted in opposite directions, each towards the population-wide values of non-admixed European and African populations. This cannot be attributed to a casual fragmentation of the genome: shifts towards or away from 0 are found significant in most cases when comparing with simulated aspPSs distributions obtained assigning a random local ancestry pattern (one-sided Wilcoxon signed-rank test, see Fig. 2, Supplementary Data 1). However, decreasing the size of the genomic portion *p*, used in computing aspPS, also decreases the directional bias following a squared root relation with the forenamed genomic fraction *p*. We can eliminate this effect by correcting the aspPS for $1/\sqrt{p}$, at the expense of an increased standard deviation for small genomic fractions. Nevertheless, including these “corrected” aspPS in parameter fitting proved to disrupt trait predictability, thus suggesting to bypass this correction. See Supplementary Note 1 for a more detailed description of this correction and related data.

These preliminary results on admixed genomes showed promising evidences for the usage of aspPS in dissecting the contributions of two ancestries towards the total PS. We therefore investigated the possibility to predict the phenotype for the studied individuals, when reliable effect sizes are available for at least one of the two ancestries.

### Partial PS predictivity in uniform genomes

**Fig. 3:**
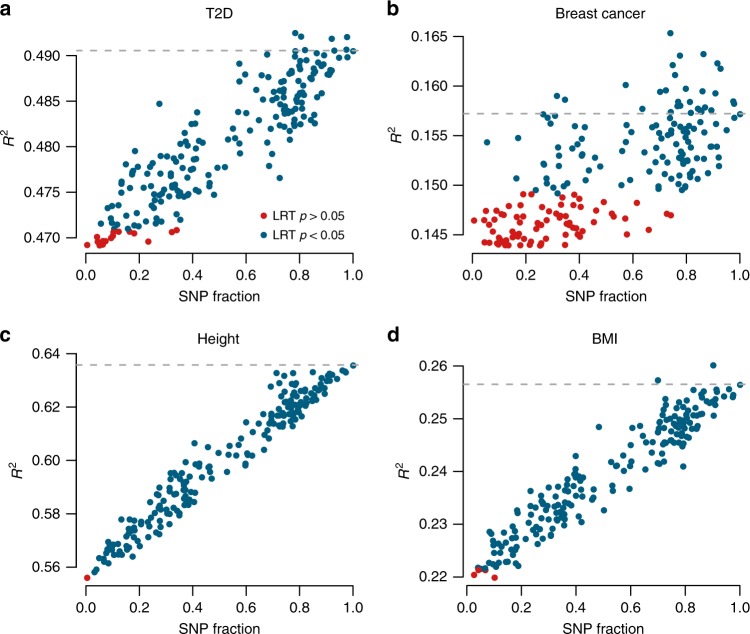
pPS predictivity. We plugged in four trait prediction models pPS obtained with genomic subsets of variable sizes (the same resulting from local ancestry analysis in our admixed individuals), in a non-admixed sample set derived from EstBB. Each point represents the performance of a different subset of the genome, applied to all individuals in the population; on the horizontal axis is reported the fraction of genomic SNPs included in each subset, while the vertical axis represents its predictivity, expressed in *R*^2^ (for binary traits we used Nagelkerke *R*^2^). Red dots represent pPS not significantly improving the base model without PS (*p* > 0.05, likelihood ratio test). The dashed line represents the total PS predictivity. (**a**) Type 2 diabetes, (**b**) breast cancer, (**c**) height, (**d**) BMI.

Before introducing differential ancestry effects in our system we tested whether a PS computed on a partial genome (pPS) can be used as proxy for the total PS and as predictor of the expressed phenotype. We relied on genomes for which phenotypes (T2D, breast cancer, BMI, height) and total PS were both available: the Estonian Biobank^31^ (EstBB). We mimicked a history of non-European admixture in EstBB samples by applying onto them the local ancestry patterns resulting from the analysis in the previous section. Importantly, even if it has been shown that PS performance can vary even among European populations^15,16^, we do not expect macroscopic inefficiencies adopting available GWAS results in Estonians^12,18^. We therefore investigated whether the pPS could be used to obtain a significant improvement in the phenotype prediction, compared to the baseline linear model calculated with no genetic information. For all traits, we show that pPS calculated with even a small portion of available genome are capable of significantly improving the phenotype prediction accuracy, to an extent that depends on the SNPs included in that portion and its size (Fig. 3, likelihood ratio test). Moreover, pPS calculated using certain local subsetting patterns tend to perform better than others, regardless of the amount of SNPs included. In essence, on the one hand we observe a macroscopic trend which broadly agrees with the hypothesis that a given genomic subset contributes additively to the phenotypes studied. On the other hand, each subsetting pattern has its own phenotype prediction performance, which we can estimate by using that particular pattern on a set of control individuals as shown above. These results suggest that in individuals where a fraction of the genome is missing or assigned to another ancestry (as in the case of Fig. 2), the pPS can be used as a proxy for the actual PS.

### AspPS in admixed genomes from UK Biobank

We then moved on testing the conclusions drawn from EstBB on the UK Biobank^27^ (UKBB), particularly focusing on the samples for which at least two major ancestry components could be detected, and for whom phenotypes were available. We used the first six principal components to select 5000 samples from the core of European UKBB individuals^27^, and to define 34212 samples as genetically non-European and putatively admixed based on their distance from the main unadmixed core (Supplementary Fig. 4a). We further explored the ancestry makeup of these individuals through an ADMIXTURE^32^ analysis, projecting the individuals onto the allele frequency spectrum inferred by the software for a set of 1000 Genomes samples, for which global ancestry and country of origin is known (Supplementary Fig. 4b). By this way we confirmed the admixed/unadmixed nature of the non-European samples selected from the UKBB and we further labeled the observed major ancestry components. We divided the selected UKBB samples into genetically “African”, “East Asian”, “European” and “Admixed” individuals, based on their PCA and ADMIXTURE scores, and used the former three groups as sources to perform ancestry deconvolution on the latter, see Supplementary Table 2 for sample sizes.

**Fig. 4:**
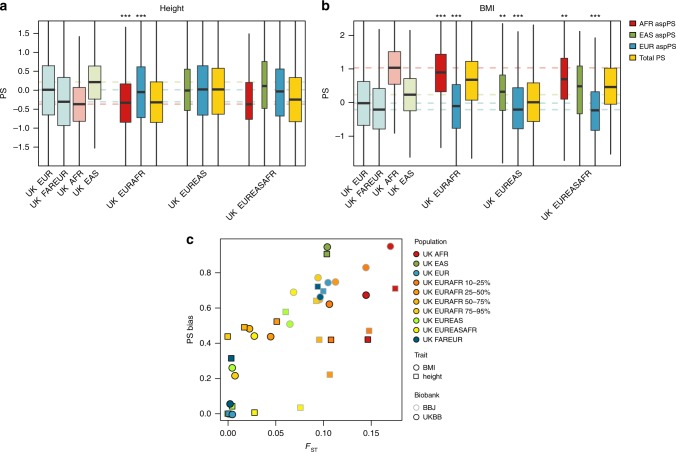
Population-wide PS and aspPS in UKBB admixed individuals. PS distributions for four reference populations (pastel colors), three admixed populations (yellow) and their relative ancestry specific partial PS (red, blue, green). Reference population medians are represented with dashed lines. The width of the boxplots is proportional to the median size of the ancestry fraction used to compute each aspPS. Two different PS are shown: (**a**) height^29^ and (**d**) BMI^29^. Significant differences with randomly assigned ancestral components are encoded as: *: *p* ≤ 0.05, **: *p* ≤ 0.005, ***: *p* ≤ 10^−5^ (one-sided Wilcoxon signed-rank test). Sample sizes and exact P-values are reported in Supplementary Data 1. For each distribution, the box represent the interquartile range (IQR = *Q*_3_−*Q*_1_), the line across the box indicate the median, the whiskers extend to the most extreme data points within *Q*_1_−1.5IQR and *Q*_3_ + 1.5IQR, outliers are omitted. (**c**) PS bias, defined as mean PS difference not explained by trait difference, is compared with *F*_*ST*_ against the reference population. All populations extracted from UKBB are represented, showing for UK EURAFR the fraction of European ancestry, UK EUR is the reference population for UKBB-based PSs, while UK EAS is the reference for BBJ-based PSs. EUR indicates european descent, EAS east asian descent, AFR african descent; combinations indicate admixed samples. FAREUR indicates Europeans far from the UKBB core.

The ancestry deconvoluted UKBB admixed samples were then grouped according to their inferred ancestry fractions (Supplementary Table 2), and pPS calculated for height and BMI traits. We chose to explore only these two phenotypes due to the shortage of clearly labeled T2D or Breast Cancer cases in our admixed UKBB cohort, and because of the higher precision achievable in discerning genetic contribution to continuous traits. Similarly to the Egyptian, Ethiopian and ASW case, aspPS on the European component of the UKBB admixed samples recovered a distribution of scores within the European ranges. Conversely, PS on the non-European component of the UKBB samples shows non-zero median, as shown in Fig. 4a, b, (see also Supplementary Data 1). By correcting the population-level PS with the real phenotypic deviation, we were able to quantitatively describe this directional bias, which showed a suggestive correspondence with the *F*_ST_ distance between each Non-European group and our reference set of European descent, see Fig. 4c.

Additionally, the same effect was observed in PSs derived from Biobank Japan^33,34^ (BBJ) and standardized against our sample set of East Asian descent from UKBB (Supplementary Fig. 5). Here East Asian aspPSs shifted towards 0 and PS bias was proportionate with *F*_ST_ against the East Asian reference set (Fig. 4c).

### AspPS predictivity in admixed genomes

**Fig. 5:**
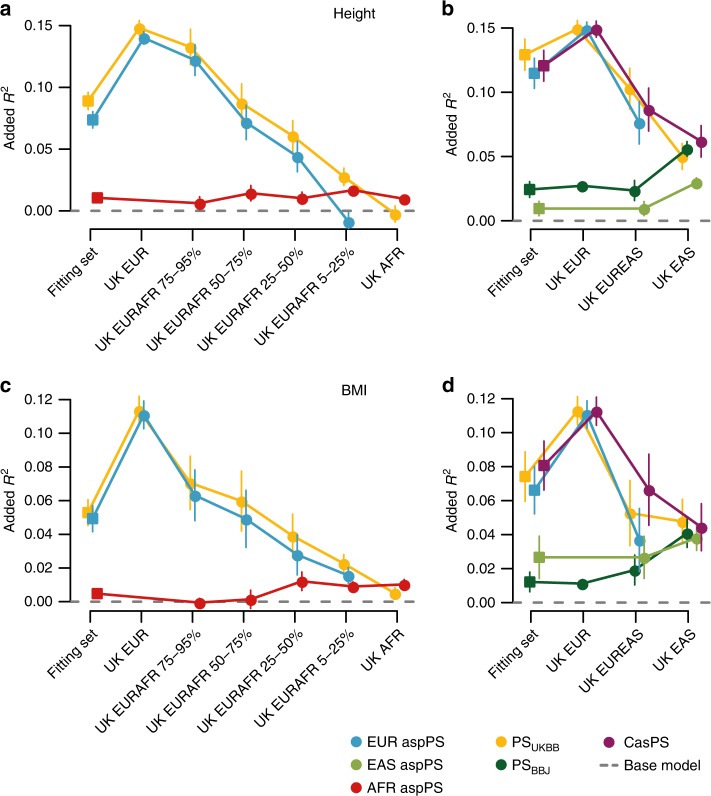
Predictivity in admixed genomes. Each plot shows the improvement in *R*^2^ when adding a PS to the base, non-genetic model. The line color depicts which PS configuration has been used: traditional total PS (PS_UKBB_ or PS_BBJ_ according to the Biobank of origin), partial ancestry specific PSs or combined ancestry specific PS. Dots represent the realized *R*^2^ improvement in each set without resampling, while bars represent standard deviation derived from n=5000 bootstrap replications. **a** Added *R*^2^ for height in UKBB samples with admixed African and European ancestry, no casPS was available. **b** Added *R*^2^ for height in UKBB samples with admixed East Asian and European ancestry. **c** Added *R*^2^ for BMI in UKBB samples with admixed African and European ancestry; no casPS was available. **d** Added *R*^2^ for BMI in UKBB samples with admixed East Asian and European ancestry. EUR indicates european descent, EAS east asian descent, AFR african descent; combinations indicate admixed samples.

We then tested the phenotype predictivity of aspPS on admixed genomes extracted from UKBB. We fitted a model on a joint, balanced set of admixed and unadmixed European individuals, and adopting as predictor a) the traditional PS or b) aspPSs, in addition to non-genetic covariates and the global ancestry proportion. This last predictor was introduced to account for predictivity gained simply scaling the total PS with the ancestry fraction: according to our simulations (Supplementary Fig. 1), this approach would be enough to recover predictivity loss due to PS directional bias alone. As shown in Fig. 5 (blue and yellow lines) aspPS computed on the European ancestral component, although comparable, never outperform the predictivity of the total PS for the admixed individuals. As anticipated by our simulations (Supplementary Fig. 1), this is expected when (A) transferability to the non-European ancestry of the trait-SNP associations discovered in Europeans is greater than zero and (B) the directional bias in PS introduced by the non-European genetic component is moderate. Nonetheless, this result confirms that the genomic portion discarded by the local ancestry deconvolution only adds, indeed, a negligible amount of information: this is further substantiated by the poor performance achieved by a control where the local ancestry patterns were randomly assigned by design, see Supplementary Fig. 6.

As anticipated by our simulations, the main advantage of computing aspPS over simple PS lies in the possibility of combining multiple aspPS into a single, global, ancestry-informed PS. We therefore focused on samples for which at least two aspPS could be computed (i.e. individuals with East Asian and European ancestral components), thanks to the availability of European- and East Asian-derived trait-SNP associations, from UKBB and BBJ respectively. When adding together the two aspPS weighted for ancestry proportions, to generate a combined ancestry specific PS (casPS), we show that the phenotype predictivity outperforms both aspPS and at least one of the total PS (Fig. 5b, d purple line). Again, this is not achieved with incorrect local ancestry inference (Supplementary Fig. 6b, d). Different fitting sets were adopted: no macroscopic differences were evident when fitting the model parameters on either admixed samples alone or European samples only, see Supplementary Fig. 7. Lastly, we compared models including casPS with the traditional total PS, performing a Vuong’s closeness test to detect which one fared better when predicting traits in admixed samples. For BMI, our approach resulted significantly closer to the real data than a traditional PS using either BBJ or UKBB associations, whereas for height the PS derived from UKBB still performed better (Supplementary Table 3).

## Discussion

We showed that in admixed individuals aspPS can recover an unbiased distribution of PS by masking out the spurious shifting effect introduced by genomic segments derived from deeply divergent populations. This enables the assessment of a sample’s PS within a European reference set when dealing with PS calculated using European genetic effect sizes (Figs. 2, 4). The aspPS approach can be transferred to samples where a fraction of the genome is simply missing (pPS). On the other hand our approach allows the evaluation of the pPS predictivity (Fig. 3) on an individual-basis, hence enabling a per-sample evaluation of the reliability of the phenotype prediction. Our simulations and analyses on real admixed individuals from the UKBB show that, depending on the cross-population transferability of the adopted trait-SNP associations, the aspPS we introduced here provides an alternative mean of assessing an admixed individual’s PS and, when multiple aspPS are combined in a casPS, the casPS predictivity outperforms both aspPS and at least one of the total PS scores (European or East Asian total PS, in our examples). Intuitively, and according to our simulation, casPS performance is enhanced by the lack of PS transferability across ancestries and if the Non-European PS performs at least as well as the European one on Non-European sample sets. Our results show that this is not the case, since PSs calculated using BBJ (East Asian) associations perform worse on East Asians than PSs calculated using UKBB associations, regardless of the adopted fitting (Fig. 5 and Supplementary Fig. 7). The difference in overall performance between the two PSs on the respective unadmixed individuals, possibly due to differences in statistical power between the underlying GWAS, might be the reason why casPS for height did not show a significant improvement over the total PS calculated using UKBB trait-SNP associations (Fig. 5b), although it did compared to total PS computed with weights from BBJ. In either cases, however, we maintain that aspPS and casPS are preferable measures than the simple PS, despite their comparable predictive performance, due to the fact that their features are all based on the correct pairing of SNP-trait associations and local ancestry. In conclusion pPS, aspPS and casPS are good predictors of both the PS and of the underlying phenotypes and provide a crucial step toward the extension of personalized and predictive healthcare to individuals of admixed ancestry, where at least part of the background is still of European origin or from a population with a solid GWAS groundwork. Future directions will include the extension of the method to individuals where European ancestry does not constitute a considerable genomic portion. To this extent, a better understanding of other ancestry-specific genetic effect sizes is needed. Such knowledge will provide the final step to the refinement of a tool, which under an additive model, can incorporate all aspPS to produce combined ancestry specific Polygenic Scores (casPS).

## Methods

### Genetic data

We took advantage of a dataset of 220 modern Ethiopians and Egyptians jointly called with a subset of the 1000 Genomes Project^26^ from Pagani et al.^25^, adding African-Americans belonging to the “ASW” population from 1000 Genomes Project^26^, and adding other modern populations from the 1kg project as reference : “CEU”, “IBS”, “TSI”, “YRI”, “LWK”, “CHB”. All Ethiopians with the exception of Gumuz were merged and labeled as “ETHIOPIA”. To test pPS predictivity we relied on two different datasets, one extracted from UK Biobank^27^,accessed under Project #17085, and one extracted from Estonian Biobank^31^, accessed with Approval Number 285/T-13 obtained on 17/09/2018 by the University of Tartu Ethics Committee.

### Samples selection from Biobanks

To test T2D, height, BMI in EstBB we used the same set of 1923 individuals with 942 cases of diabetes, obtained by removing samples used in training T2D PS in Läll et al.^28^ and samples genotyped by sequencing. To test breast cancer with PS from Michailidou et al.^30^ we used a set of 908 women including 308 cases, removing prevalent samples used in training PS in Michailidou et al.^30^. All these sets were filtered removing samples with relatedness of 2nd degree and higher. To test predictivity in UKBB we first removed all samples with relatedness of 3rd degree and higher, those for which relatedness could not be computed and those present in the UKBB GWAS training set^29^. Then we used a method adapted from Neale Lab (https://github.com/Nealelab/UK_Biobank_GWAS) to draw ellipses in the space defined by first 6 PCs pre-computed by the UKBB workgroup, thus selecting individuals which were (a) closer than 5 and (b) farther than 15 cumulated standard deviations with respect to the UKBB GWAS training set: this defined a genetically “European” and a “non-European” sample sets (Supplementary Fig. 4a). The “European” set was randomly downsampled to 5000, defining “UK EUR”. We performed a preliminary global ancestry analysis with ADMIXTURE^32^ at *k*=5 (Supplementary Fig. 4b) on the “non-European” set in order to select LAD sources, using only markers available from chromosome 1 and projecting the samples onto the allele frequencies (P file) obtained by running the 2305 1000 Genomes Project samples^26^ under the same parameters. We also discarded samples which showed cumulative South Asian or Native American ancestry higher than 20%. We further segmented genetically non-European samples with the global proportions obtained from the LAD by assuming a threshold of 5% in order to define presence/absence of a certain ancestry in an individual, thus defining “UK AFR”, “UK EAS”, “UK EURAFR”, “UK EUREAS”, “UK EUREASAFR”. “UK FAREUR”, most likely composed by South Europeans and West Asian individuals, was defined by downsampling to 5000 a group of samples inferred to be more than 95% European by LAD but coming from “non-European” PC space. See Supplementary Table 2 for sample sizes of all UKBB-derived sets.

### Local ancestry analysis

To perform the LAD we adopted ELAI^24^, a two-layer HMM software designed to learn the structure of local and long distance ancestral haplotypes. We adopted two combinations of 72 samples equally distributed among CEU, TSI, IBS and GUM, LWK, YRI, respectively, as sources for West-Eurasian and African segments for the Egyptian, Ethiopian and ASW samples. Concerning UKBB samples, to minimize batch effects we resorted to internally available source samples. To this extent, 100 samples were extracted from the “UK EUR” set to act as European sources, while as African and East Asian sources we extracted the 100 with the highest appropriate ancestry fraction according to ADMIXTURE. The admixture generations parameter was set as 100 for Ethiopians, 30 for Egyptians, 6 for ASW and 10 for UKBB samples. Each time we used 20 EM steps and performed 10 runs, of which we took the average. We assigned each SNP to the ancestry with the highest estimated allelic dosage, provided it reached a threshold of 0.9, or labeled it as unknown otherwise. In all cases we used phased data, treating haplotypes as independent.

### Summary statistics for PSs

To compute PSs we adopted summary statistics from a collection of studies listed in Supplementary Table 1. Summary statistics were filtered removing palindromic SNPs and those with MAF < 0.01, then clumped applying PLINK 1.9^35^, with parameters: -clump-r2 0.05 -clump-p1 0.5 -clump-p2 1 -clump-kb 1000. The best set of SNPs was selected by determining the best *P*-value cutoff with PRSice^36^. Both clumping and cutoff selection were run on the reference set in Supplementary Table 1. Summary statistics for T2D were taken as they are and only clumping was run on the ones for Breast Cancer.

### Computing pPS

We formulated a statistic that estimates the total Polygenic Score (PS) using only a part of the genome: a “partial Polygenic Score” (pPS). Its desired key properties are: (A) being unbiased with respect to the fraction of genome used to compute it and (B) being robust to fluctuations in variance due to the different regions of the genome used to compute pPS.

A common^12,28^ definition of a raw PS for the individual *j* is the sum of its alleles weighted by their effect sizes:

2 $${S}_{j}=\mathop{\sum }\limits_{i=1}^{{N}_{{\rm{V}}}}{\beta }_{i}{x}_{ij},$$

where *x*_*ij*_ is the allelic state at site *i*, individual *j*, *β*_*i*_ is the associated effect size and *N*_V_ is the number of variants used to compute it. An equivalent alternative is to compute the mean, instead of the sum:

3 $${\overline{x}}_{j}=\frac{1}{{N}_{{\rm{V}}}}\mathop{\sum }\limits_{i=1}^{{N}_{{\rm{V}}}}{\beta }_{i}{x}_{ij}.$$

In order to compute partial PS we only consider a subset of variants of the genome, with size *N*_S_ (*N*_S_ < *N*_V_); we can therefore define a raw pPS as

4 $${\overline{x}}_{j}^{\prime}=\frac{1}{{N}_{{\rm{S}}}}\mathop{\sum }\limits_{i=1}^{{N}_{{\rm{S}}}}{\beta }_{i}{x}_{ij}.$$

We then devised a standardized pPS using a Z-score of the raw pPS, as is common:

5 $${{\rm{pPS}}}_{j}=\frac{{\overline{x}}_{j}^{\prime}-{\mu }_{\overline{x}^{\prime} }}{{\sigma }_{\overline{x}^{\prime} }},$$

where ${\mu }_{\overline{x}^{\prime} }$ and ${\sigma }_{\overline{x}^{\prime} }$ are, respectively, mean and standard deviation of the $\overline{x}^{\prime}$ statistic, computed across all *N*_I_ individuals of a reference population and using only a subset of variants of the genome, with size *N*_S_. The traditional standardized PS is a limit case of such formula where *N*_S_ = *N*_V_. We independently computed PS for phased haplotypes, using their own local ancestry pattern in case of aspPS, and subsequently merged the results from the two haplotypes of the same individual.

### Population-wide aspPS analyses

The true aspPSs were compared with aspPSs of the same individual produced with an incorrect local ancestry pattern with a one-sided Wilcoxon signed-rank test. This control was obtained shuffling the local ancestry patterns among individuals of the same population, excluding the correct pattern-individual matching. The test side was always towards 0 for the reference population and away from 0 otherwise.

PS bias was estimated as mean PS minus mean trait measure, after standardizing both quantities against the reference population. *F*_ST_ with PS reference populations was estimated using vcftools 0.1.16^37^ and the option -weir-fst-pop <P1> -weir-fst-pop <p2>, where p1 and p2 are all the analyzed groups. In all analyses described in this section and in the boxplots in Figs. 2, 4 and Supplementary Fig. 5, we removed aspPS obtained from less than 10% of the genome.

### pPS Predictivity in uniform genomes

As a local ancestry pattern is essentially a subset of the genome obtained superimposing a mask onto the genomic fragments of other ancestries, we could apply the same subsetting mechanism to other genomes, even if non-admixed. We chose randomly 200 European local ancestry/subsets from our admixed sets. Each of the drawn subsets (masks) obtained in the local ancestry analysis was applied to the whole EstBB dataset to obtain a pPS distribution and predicted *R*^2^ was used as measure of predictivity. For binary traits we adopted a logistic model and the Nagelkerke *R*^2^.

### aspPS predictivity

We first fitted a glm model for each different PS configuration, each time including as covariates sex, age, age^2^, age^3^, genotyping batch and European SNP fraction: these alone define a “base” model. We adopted different sets for this fitting step, including: (a) a balanced set of UK EUR plus admixed sets, (b) a balanced set only including admixed sets, (c) UK EUR as is, (d) a balanced set of UK EAS plus admixed sets, (e) a balanced set of all populations, see Supplementary Fig. 7. We then measured *R*^2^ on specific sample sets by predicting their phenotypes with the model fitted above and assessed standard deviation through 5000 bootstrap replications (boot package from R Software (https://www.r-project.org)). Added *R*^2^ is defined as realized *R*^2^ minus base model *R*^2^. Comparisons of nested and non-nested models were performed with likelihood ratio test from lmtest and Vuong test from nonnest2 R packages, respectively. When testing in EstBB we used the genotyping platform as covariate in place of genotyping batch. The incorrect local ancestry was defined as in “Population-wide aspPS analyses”.

### Reporting summary

Further information on research design is available in the Nature Research Reporting Summary linked to this article.

## Supplementary information

Supplementary Information

Description of Additional Supplementary Files

Supplementary Data 1

Reporting Summary

## Data Availability

The datasets analyzed during the current study are publicly available and can be accessed from the following repositories: data from 1000 Genomes Project at ftp://ftp.1000genomes.ebi.ac.uk/vol1/ftp/; data from Pagani et al.^25^ at https://ega-archive.org/datasets/EGAD00001003296; data from UK Biobank at https://biobank.ndph.ox.ac.uk/showcase/ (accessed under Project #17085); data from Estonian Biobank at https://genomics.ut.ee/en/access-biobank (accessed with Approval Number 285/T-13 obtained on 17/09/2018 by the University of Tartu Ethics Committee). GWAS summary statistics can be accessed at https://www.nealelab.is/uk-biobank and http://jenger.riken.jp/en/result for UKBB and BBJ respectively.

## Code availability

Code for analyses performed in this paper is accessible at the repository: https://bitbucket.org/dmarnetto/ancestry-specific-partial-ps.
