# Supplementary material for: Ancestry deconvolution and partial polygenic score can improve susceptibility predictions in recently admixed individuals
Source: Nat Commun. 2020 Apr 2;11:1628. doi: 10.1038/s41467-020-15464-w (PMC7118071; doi:10.1038/s41467-020-15464-w)
Supplement: Supplementary file 1 — Supplementary Information [file 41467_2020_15464_MOESM1_ESM.pdf]

Ancestry deconvolution and partial polygenic score can improve susceptibility predictions in recently admixed individuals

Supplementary Information

Davide Marnetto *et al.*

Supplementary Note 1
Partial PS bias for decreasing portions of the genome

Partial PS decrease their directional bias when computed on decreasing portions of the genome, see Supplementary Figure 8. Correcting the denominator with $\sqrt{p}$ recovers this, at the cost of increasing the standard deviation. This might be seen as an effect of adding more and more "bias-causing" genome, while the corrective power of $\sqrt{p}$ can be explained with the following paragraph.

We cannot think of an individual genome as a single indivisible unit, because we want to compute statistics for parts of individuals. On the contrary, we might think of an individual, and of a subset of its genome, as a sample of weighted alleles, and of its raw pPS as a sample mean $\bar{x}'$. Consequently, the normalizing terms in the Z-score standardization of this sample mean should be the mean and the standard deviation of the sample means, $\mu_{\bar{x}'}$ and $\sigma_{\bar{x}'}$ (i.e. the mean raw pPS across individuals and its standard deviation). We thus introduce a dependency on the number of variants that we include in our genomic subset: in fact the standard deviation of the mean for random samples is estimated as $\sigma_{\bar{x}} = \frac{\sigma}{\sqrt{n}}$ where n is the sample size (in our notation N_{V} for PS and N_{S} for pPS). To control for this dependency we add the square root of the SNP fraction $p = \frac{N_{\mathrm{S}}}{N_{\mathrm{V}}}$ as correction factor to $\sigma_{\bar{x}'}$:

$$pPS = \frac{\bar{x}'_j - \mu_{\bar{x}'}}{\sqrt{p} \cdot \sigma_{\bar{x}'}} \tag{1}$$

Even if individuals as samples of weighted alleles cannot be considered random, this approach provides a constant Z-score denominator, as showed in Supplementary Figure 9, thus making comparisons possible by removing the dependency on the SNP fraction.

To generate Supplementary Figure 9, we simulated a dataset with 100 individuals and 1000 variants. Minor allele frequencies were simulated as normally distributed with $\mu = 0.2$ and $\sigma \in \{0.05, 0.5\}$, then trimmed to 0 and 1 if exceeding. Variant weights were simulated as normally distributed with mean $\mu = 0$ and $\sigma = 1$. Boxplot distributions were generated sampling 100 subsets with size N_{S}.

Nevertheless, including these p-corrected aspPS in parameter fitting proved to disrupt trait predictability, see Supplementary Figure 10. In fact, poorly predictive aspPS with larger sd, increased by such correction, cause the coefficient associated to PS to behave erratically. As a PS should essentially be a good predictor for phenotypic traits, we favoured a more tractable and predictive version, disregarding the property of maintaining a stable population-wide bias, bypassing this correction.

Supplementary Table 1: Summary statistics adopted in this study

Label	Trait	SNPs after filters	P-value threshold	Source	Reference populations
T2D	Type 2 Diabetes	7500	0.5*	Läll *et al.* [1]	EUR, EstBB
Breast Cancer	Breast cancer	44246	0.5*	Michailidou *et al.* [2]	EUR†, EstBB
height	Height	35246	0.05	UKBB[3]	EUR, EstBB, UK EUR†‡
$\text{height}_{\text{BBJ}}$	Height	1654	5e-05	BBJ[4]	UK EAS†‡
BMI	Body Mass Index	92832	0.4	UKBB[3]	EUR, EstBB, UK EUR†‡
BMI_{BBJ}	Body Mass Index	11416	0.05	BBJ[5]	UK EAS†‡

The P-value threshold was defined through PRSice, based on the summary statistics obtained from Sources. The Reference populations were used to standardize PS in the respective datasets. *: threshold taken as is from Source; †: reference used to perform clumping; ‡: reference used to perform PRSice analyses, all of them.

Supplementary Table 2: Sample sizes of subsets from UKBB used in this study

PCA-based selection set	label	size
Non-European	UK NONEUR	34212
European	UK EUR	5000
TOTAL		39212

ADMIXTURE-based selection set	label	size
European	UK EUR	4900
Admixed	ADMIXED	21011
African sources	S_AFR	100
East Asian sources	S_EAS	100
European sources	S_EUR	100
TOTAL		26211
DISCARDED		13001

ELAI segmentation set	label	size
Individuals of African and European descent	UK EURAFR	4930
Individuals of East Asian and European descent	UK EUREAS	618
Individuals of African, East Asian and European descent	UK EUREASAFR	245
Non-British European	UK FAREUR	5000
African	UK AFR	4093
East Asian	UK EAS	2168
European	UK EUR	5000
TOTAL		22054
DISCARDED		4157

UK EURAFR segmentation set	label	size
Individuals of African and European descent with different European proportions	UK EURAFR 5-25%	2593
	UK EURAFR 25-50%	879
	UK EURAFR 50-75%	624
	UK EURAFR 75-95%	834
TOTAL		4930

Supplementary Table 3: CasPS Vuong closeness test

trait	other model	True local ancestry pattern: CasPS fits better	True local ancestry pattern: Other fits better	Incorrect local ancestry pattern: CasPS fits better	Incorrect local ancestry pattern: Other fits better
height	base	< 2e-16*	-	< 2e-16*	-
height	PS_{UKBB}	1	< 2e-16	1	< 2e-16
height	PS_{BBJ}	< 2e-16	1	< 2e-16	1
BMI	base	4.76e-11*	-	2.32e-05*	-
BMI	PS_{UKBB}	< 2e-16	1	1	< 2e-16
BMI	PS_{BBJ}	< 2e-16	1	< 2e-16	1

One-tailed P-values from Vuong closeness test. For nested models ($*$) we report nested likelihood ratio test one-tailed P-values.

Supplementary Data 1: Sample sizes, P-values and test statistics for population-wide PS and aspPS. This table reports the sample sizes for all distributions shown in Figure 2,4 and Supplementary Figure 5. Here we report also exact pvalues from Wilcoxon signed-rank test, together with side of the test and wilcoxon statistic, for each aspPS distribution shown in the figures above.

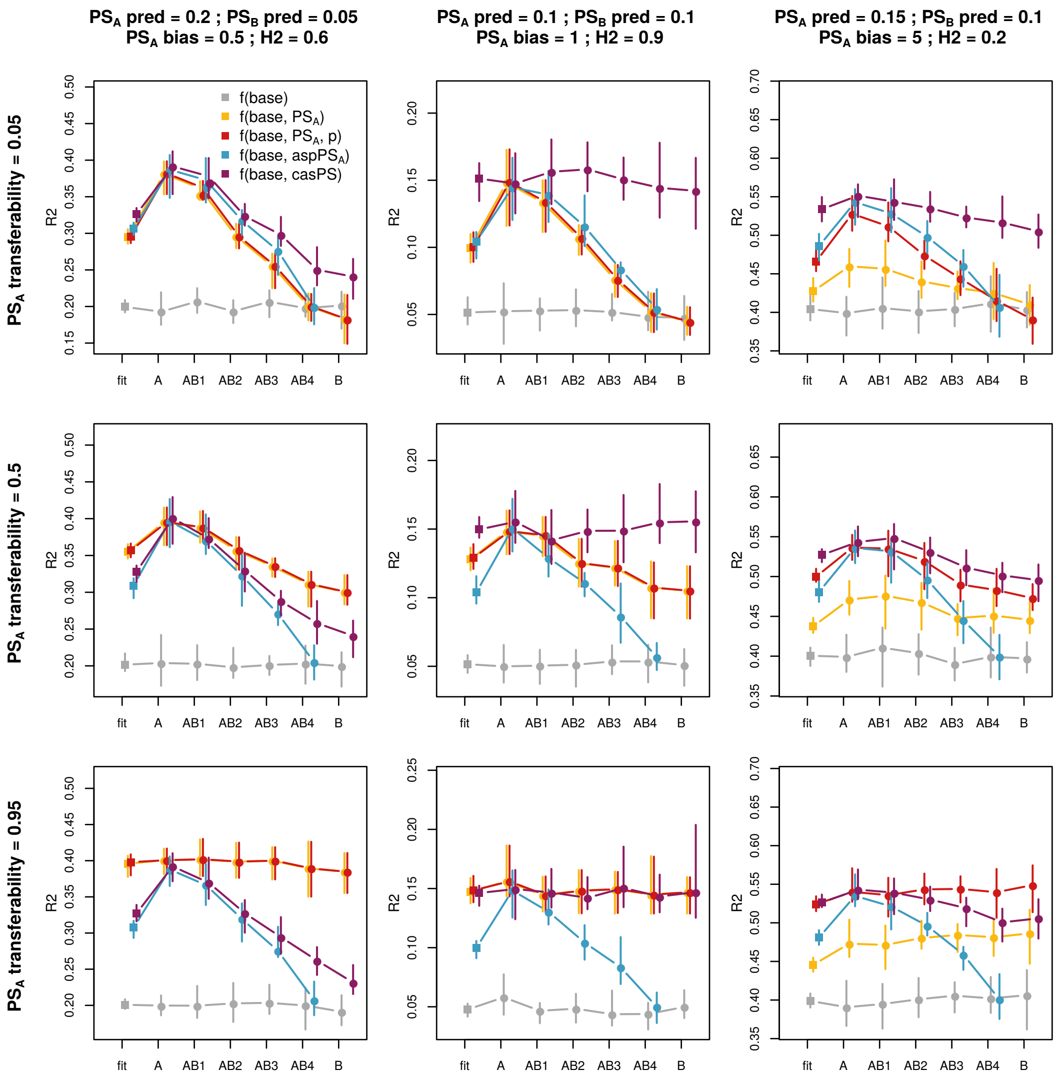

Supplementary Figure 1: Simulation results. A simulation was performed to provide expectations for our working hypothesis. An unbiased, precise and a biased, noisy PS were combined in a single value to purport admixed individuals, several values for different parameters were explored. Two populations A and B and four admixed populations AB with increasing portion of B are shown on the x axis. Each population has a size n=2500, dots and bars represent respectively mean and range over 10 independent replications of the simulation. Each line shows the predictive performance of a model including the predictors showed in legend, where PS is the PS non-biased for the population A ($\mathrm{PS_A}$), p is the proportion of A, $\mathrm{aspPSS_A}$ is the partial PS only including the portion A, and casPS is the p-weighted combination of $\mathrm{PS_A}$ and $\mathrm{PS_B}$ (PS precise, unbiased for population B). On the rows we have 0.05, 0.5 and 0.95 transferability, defined as the predictivity maintained when computing $\mathrm{PS_A}$ onto the portion B of an individual. On the columns we show three different assortments of parameters representing a case with a highly predictive $\mathrm{PS_A}$ and poorly predictive $\mathrm{PS_B}$, a case with both PS equally predictive in their own population, and a case with high directional bias.

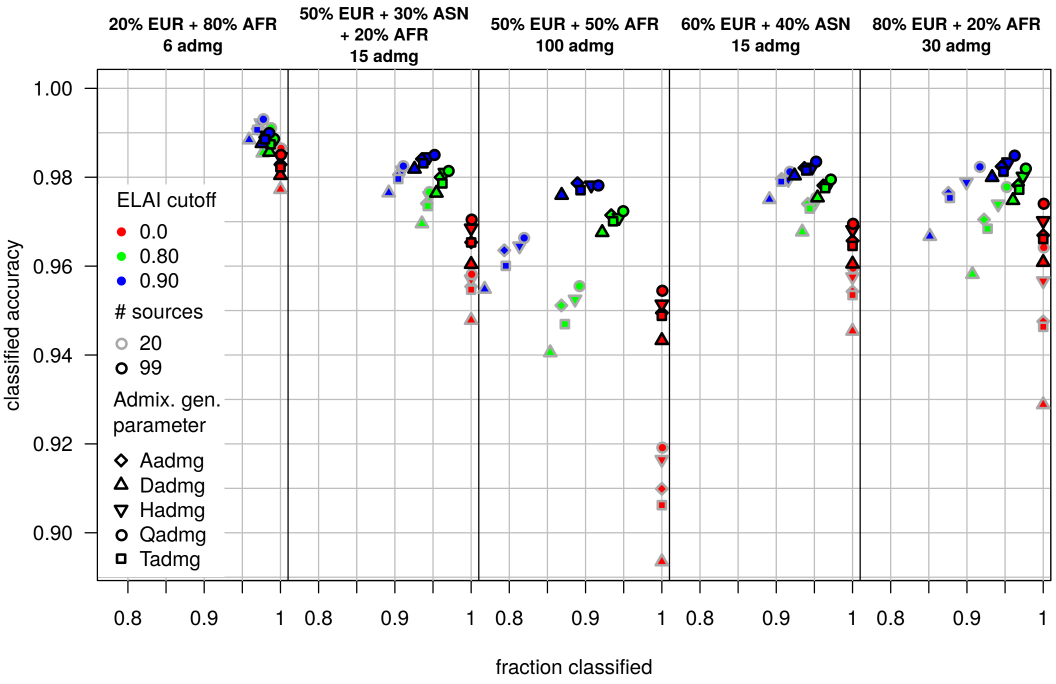

Supplementary Figure 2: ELAI accuracy analysis. We simulated a series of admixed populations of size 30 with Admix-Simu from Williams Lab (`https://github.com/williamslab/admix-simu`), using EUR, EAS, AFR from 1000 genomes project[6] as donors and inferred local ancestry using CEU,CHB,YRI[6] as sources. Different cutoffs on the ancestry dosage (colors), two different source sets (borders) and 5 different admixture generation parameters (shapes) were used. Tadmg: real admix. gen. used in simulating the population, Dadmg: double of the true admix. gen., Hadmg: half of the true admix. gen., Aadmg: an average of the three former inferences, Qadmg: a quarter of the true admix. gen.

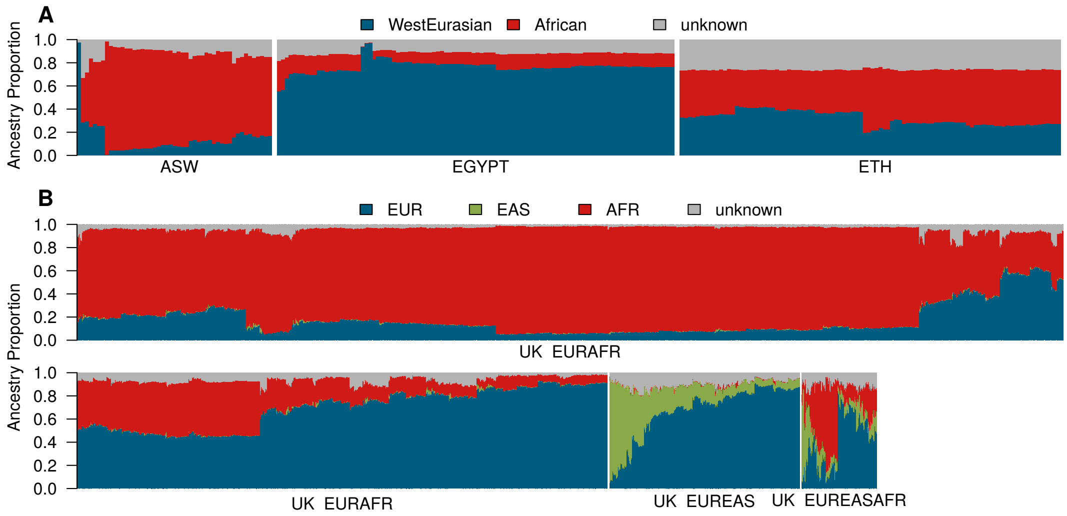

Supplementary Figure 3: Local Ancestry Assignment, for each individual, as resulting from our local ancestry analysis (ELAI). The proportion is computed as fraction of snps. **(a)** Ethiopians and Egyptians from Pagani *et al.* [7], African-Americans from 1000 Genomes Project[6]; **(b)** admixed samples from UKBB.

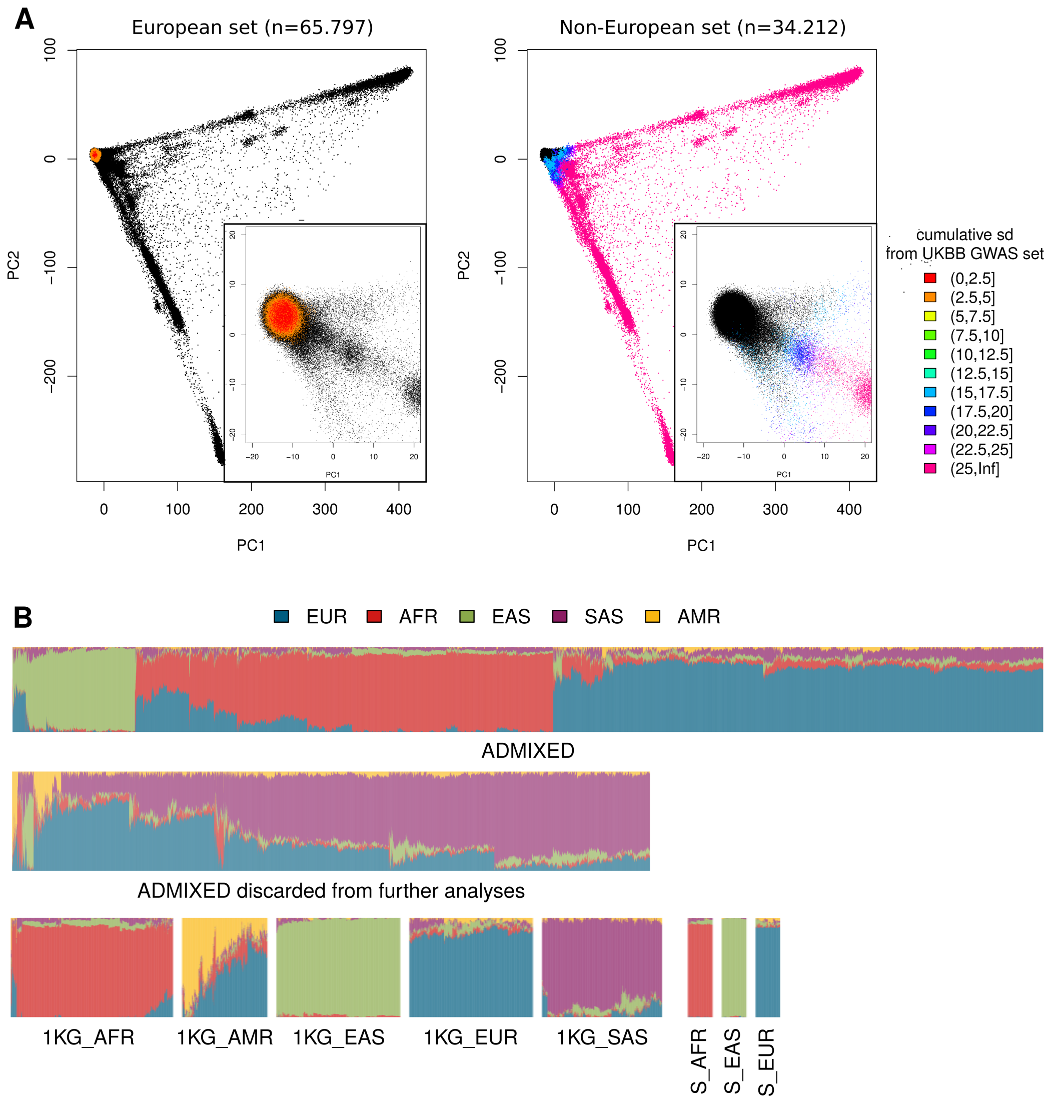

Supplementary Figure 4: UKBB admixed samples selection. **(a)** first two principal components of UKBB samples, the European and Non-European sets are highlighted. We used colors to represent distance computed on the first 6 PCs on which we designated a threshold of 5 and 15 standard deviations to define European and Non-European samples. **(b)** ADMIXTURE ancestry components for UKBB Non-Europeans and for 1000 genomes samples (K=5). The last row is magnified 5-fold.

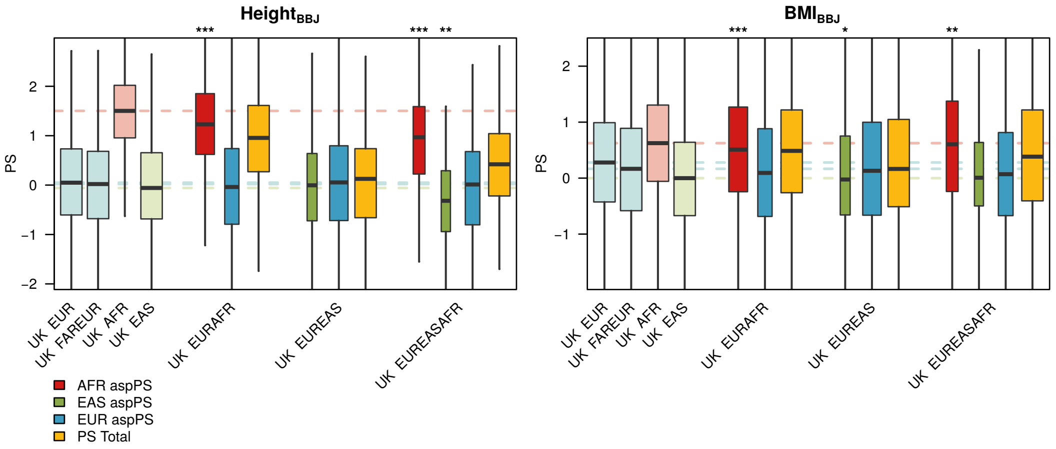

Supplementary Figure 5: Population-wide BBJ-derived PS and aspPS in UKBB admixed individuals. As Main Figure 4 but adopting PS derived from BBJ: **(a)** height[4] and **(b)** BMI[5]. PS distributions for 4 reference populations (pastel colors), 3 admixed populations (yellow) and their relative ancestry specific partial PS (red,blue,green). Reference population medians are represented with dashed lines. The width of the boxplots is proportional to the median size of the ancestry fraction used to compute each aspPS. Significant differences with randomly assigned ancestral components are encoded as: $* : p \leq 0.05$, $** : p \leq 0.005$, $*** : p \leq 10^{-5}$ (one-sided Wilcoxon signed-rank test). Sample sizes and exact P-values are reported in Supplementary Data 1. For each distribution, the box represent the interquartile range ($\mathrm{IQR} = Q_3 - Q_1$), the line across the box indicate the median, the whiskers extend to the most extreme data points within $Q_1 - 1.5\,\mathrm{IQR}$ and $Q_3 + 1.5\,\mathrm{IQR}$, outliers are omitted.

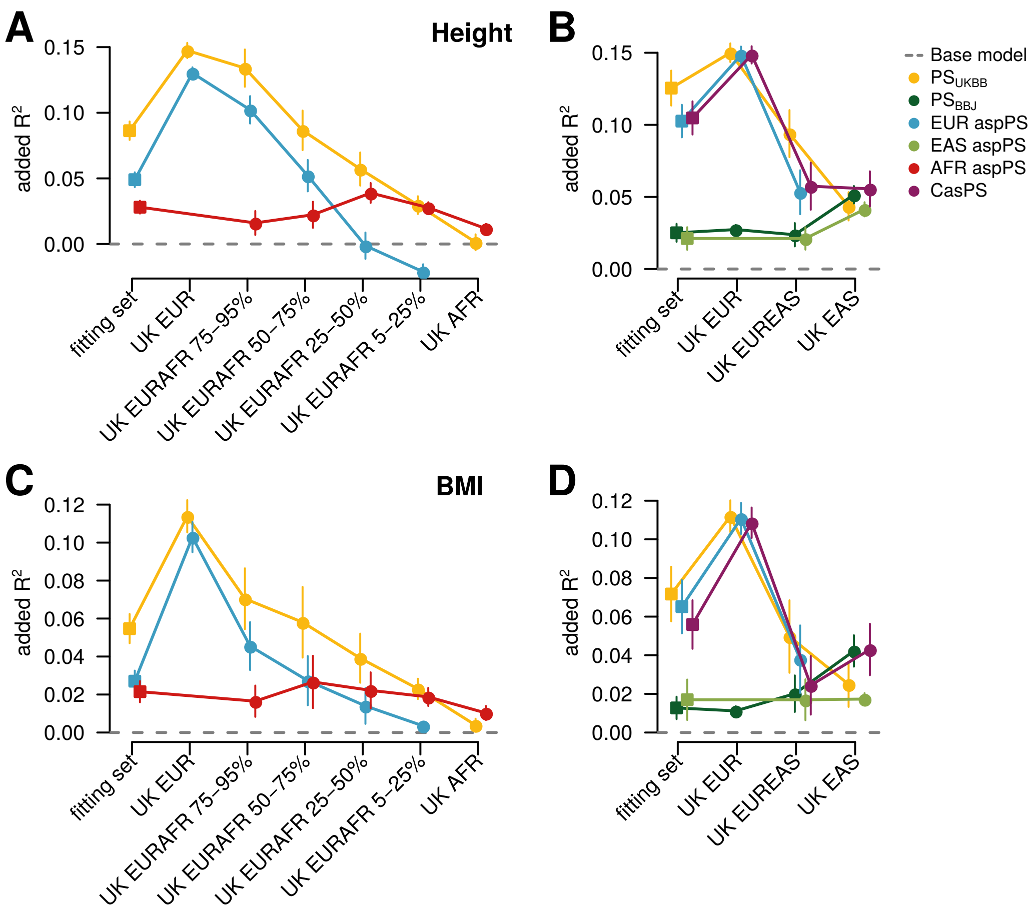

Supplementary Figure 6: Predictivity in admixed individuals, incorrect local ancestry. As in Main Figure 5 but applying an incorrect local ancestry pattern through a random shuffling between individuals and patterns of the same population. See Methods for further details. Dots represent the realized R^2 improvement in each set without resampling, while bars represent standard deviation derived from n=5000 bootstrap replications. **(a)**. Added R^2 for height in UKBB samples with admixed African and European ancestry, no casPS was available.**(b)** Added R^2 for height in UKBB samples with admixed East Asian and European ancestry**(c)** Added R^2 for BMI in UKBB samples with admixed African and European ancestry; no casPS was available. **(d)** Added R^2 for BMI in UKBB samples with admixed East Asian and European ancestry.

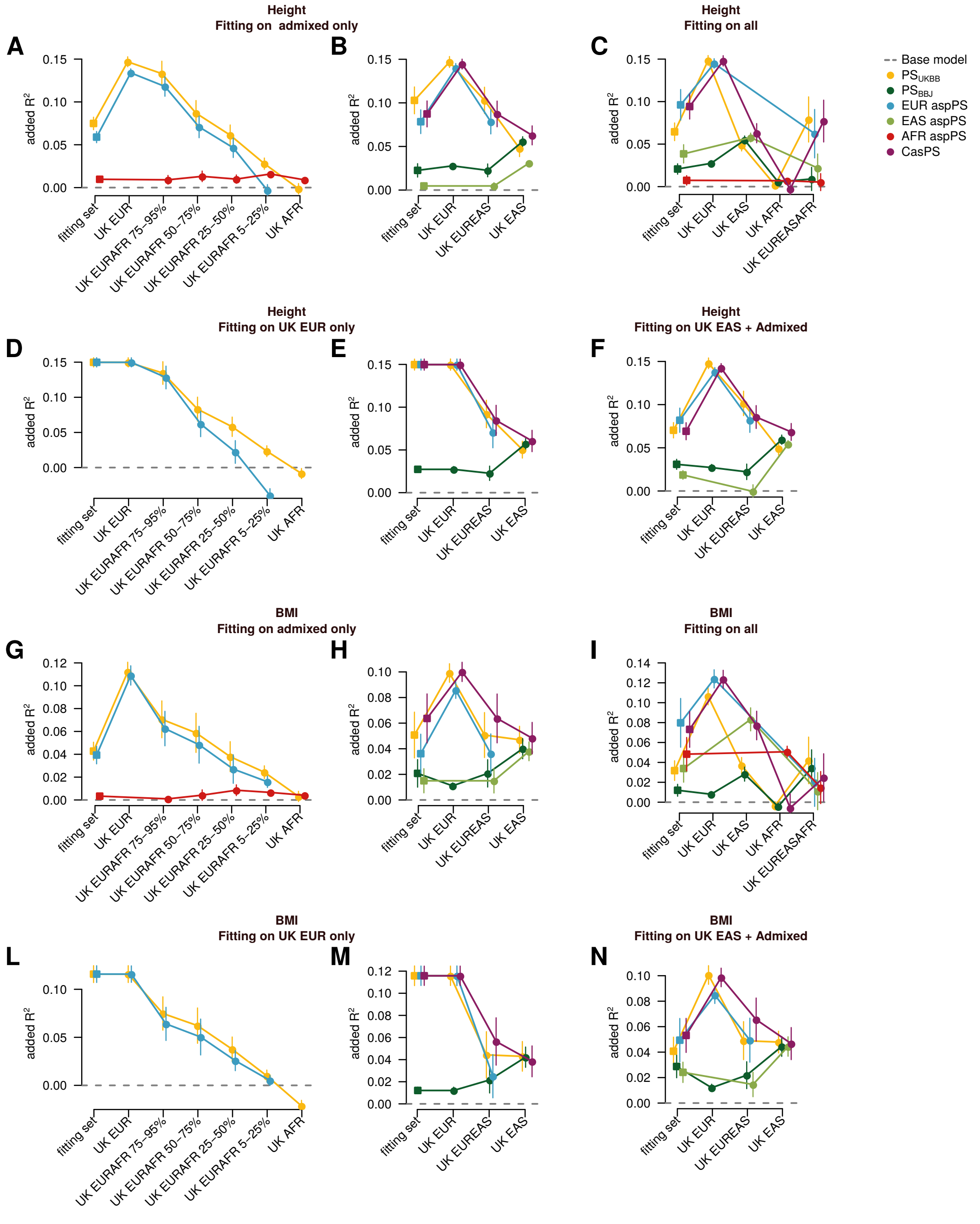

Supplementary Figure 7: Predictivity in admixed individuals, different fitting sets. As in Main Figure 5 but adopting different fitting sets. See Methods for further details. Dots represent the realized R^2 improvement in each set without resampling, while bars represent standard deviation derived from n=5000 bootstrap replications.**(a,b,g,h)**. Added R^2 for height (a,b) and BMI (g,h); fitting performed on admixed individuals only.**(c,i)** Added R^2 for height and BMI respectively, in UKBB samples with admixed East Asian, African and European ancestry; fitting performed on a balanced set including all sample sets shown. **(d,e,l,m)** Added R^2 for height (d,e) and BMI (l,m); fitting performed on UK EUR individuals only. **(f,n)** Added R^2 for height and BMI respectively, in UKBB samples with admixed East Asian and European ancestry; fitting performed on a balanced set of UK EAS and admixed individuals.

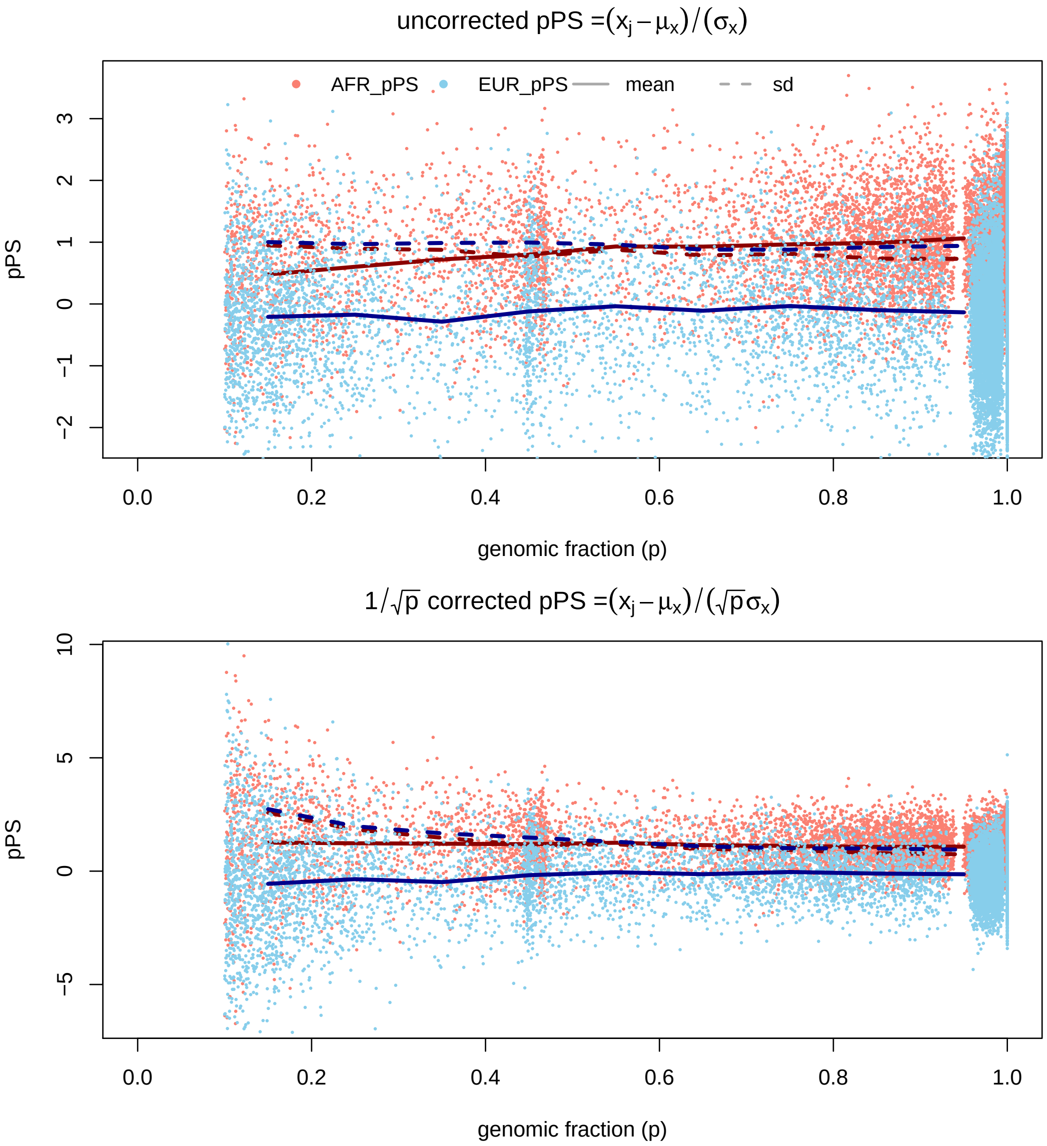

Supplementary Figure 8: Effect on pPS correcting for $1/\sqrt{p}$. (a) The uncorrected aspPS for BMI in UKBB African-European samples: the bias of African aspPS decreases with $\sqrt{p}$ but the standard deviation remains constant.**(b)** The uncorrected version, the standard deviation increases as p gets smaller but the directional bias remains constant.

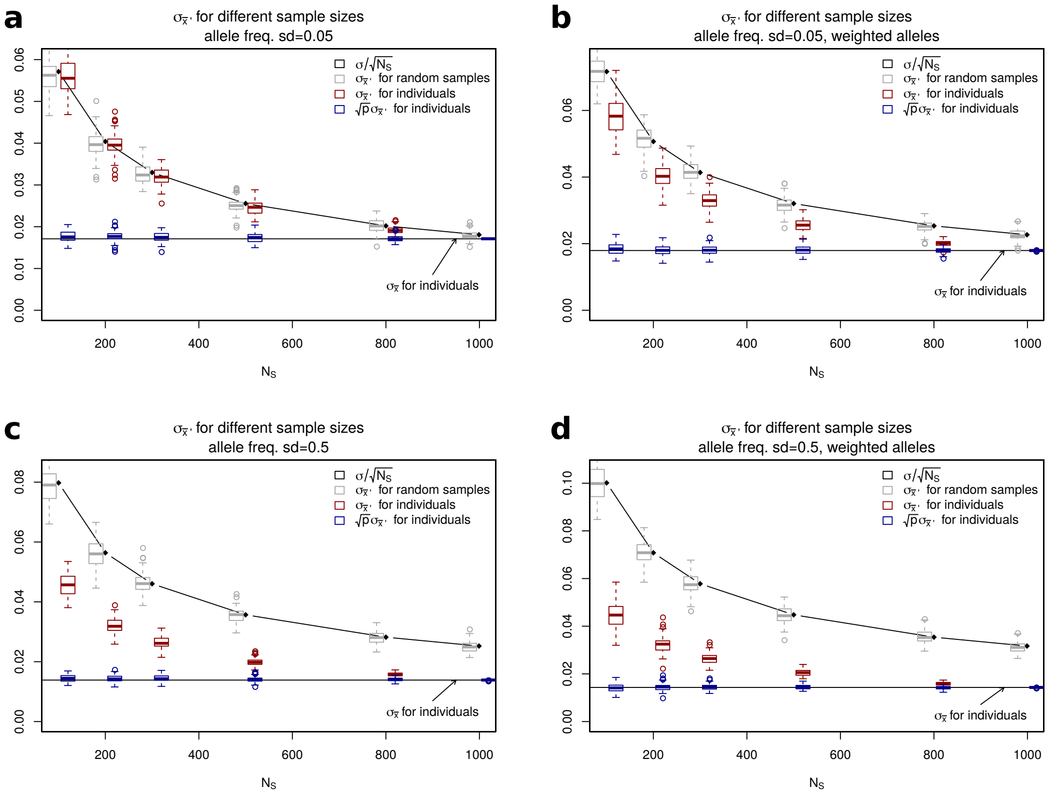

Supplementary Figure 9: Standard deviations of raw pPS according to SNP fraction. We simulated a matrix with variants on the rows and individuals on the columns. **(a,b)** Allele frequencies simulated as centered on 0.2 with sd=0.05 **(c,d)** Allele frequencies simulated as centered on 0.2 with sd=0.5 **(b,d)** variant weights applied. In these simulated settings the standard deviation of the means for random samples of different sizes N_S (grey boxes) follows the relation $\sigma_{\bar{x}} = \frac{\sigma}{\sqrt{N_\mathrm{S}}}$. By computing the standard deviation of the means for the same subset of variants/rows across individuals/columns, i.e. the standard deviation of raw pPS (red boxes), we instead observe a systematic difference with the estimate. This difference is small when allele frequencies are very similar across the genome **(a)** and becomes larger when allele frequencies are more diverse and weights are applied **(b-d)**. Nevertheless, correcting such values by multiplying $\sqrt{p}$ (blue boxes) (i.e. $\sqrt{p} \cdot \sigma_{\bar{x}'}$, with $p = \frac{N_\mathrm{S}}{N_\mathrm{V}}$), yields for any SNP fraction a stable estimate of the standard deviation obtained when all the SNPs in the genome are considered (i.e. $\sigma_{\bar{x}}$). Each box plot represent a distribution of the statistic in legend, obtained drawing n=100 random subsets of SNPs with size N_S. For each distribution, the box represent the interquartile range ($\mathrm{IQR} = Q_3 - Q_1$), the line across the box indicate the median, the whiskers extend to the most extreme data points within $Q_1 - 1.5\,\mathrm{IQR}$ and $Q_3 + 1.5\,\mathrm{IQR}$, and each outlier is represented by an individual mark.

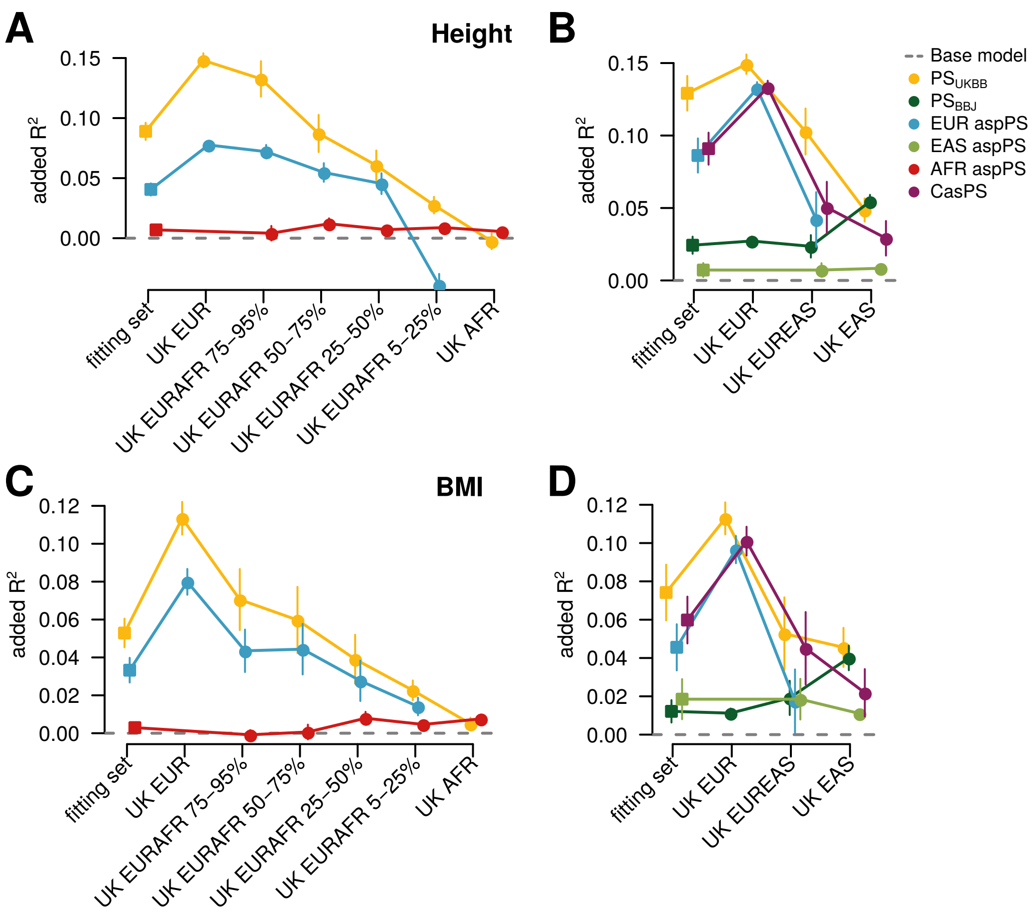

Supplementary Figure 10: Predictivity in admixed individuals, PS corrected for $1/\sqrt{p}$. As in Main Figure 5 but adopting PS corrected for $1/\sqrt{p}$. See Methods and Supplementary Note 1 for further details. Dots represent the realized R^2 improvement in each set without resampling, while bars represent standard deviation derived from n=5000 bootstrap replications.**(a)**. Added R^2 for height in UKBB samples with admixed African and European ancestry, no casPS was available.**(b)** Added R^2 for height in UKBB samples with admixed East Asian and European ancestry**(c)** Added R^2 for BMI in UKBB samples with admixed African and European ancestry; no casPS was available. **(d)** Added R^2 for BMI in UKBB samples with admixed East Asian and European ancestry.

References

1. Läll, K., Mägi, R., Morris, A., Metspalu, A. & Fischer, K. Personalized risk prediction for type 2 diabetes: the potential of genetic risk scores. *Genet. Med.* **19,** 322–329 (2017).
2. Michailidou, K. *et al.* Association analysis identifies 65 new breast cancer risk loci. *Nature* **551,** 92–94 (2017).
3. Churchhouse, C. & Neale, B. M. *UK Biobank, Neale Lab.* `http://www.nealelab.is/uk-biobank/` (2019).
4. Akiyama, M. *et al.* Characterizing rare and low-frequency height-associated variants in the Japanese population. *Nat. Commun.* **10,** 4393 (2019).
5. Akiyama, M. *et al.* Genome-wide association study identifies 112 new loci for body mass index in the Japanese population. *Nat. Genet.* **49,** 1458–1467 (2017).
6. The 1000 Genomes Project Consortium *et al.* A global reference for human genetic variation. *Nature* **526,** 68–74 (2015).
7. Pagani, L. *et al.* Tracing the Route of Modern Humans out of Africa by Using 225 Human Genome Sequences from Ethiopians and Egyptians. *Am. J. Hum. Genet.* **96,** 986–991 (2015).
