# Supplementary material for: Ancestry deconvolution and partial polygenic score can improve susceptibility predictions in recently admixed individuals
Source: Nat Commun. 2020 Apr 2;11:1628. doi: 10.1038/s41467-020-15464-w (PMC7118071; doi:10.1038/s41467-020-15464-w)
Supplement: Supplementary file 2 — Description of Additional Supplementary Files [file 41467_2020_15464_MOESM2_ESM.pdf]

Title: Supplementary Data 1

Description: This table reports the sample sizes for all distributions shown in Figure 2,4 and Supplementary Figure 5. Here we report also exact pvalues from Wilcoxon signed-rank test, together with side of the test and wilcoxon statistic, for each aspPS distribution shown in the figures above.
