# Supplementary material for: Ancestry deconvolution and partial polygenic score can improve susceptibility predictions in recently admixed individuals
Source: Nat Commun. 2020 Apr 2;11:1628. doi: 10.1038/s41467-020-15464-w (PMC7118071; doi:10.1038/s41467-020-15464-w)
Supplement: Supplementary file 4 — Reporting Summary [file 41467_2020_15464_MOESM4_ESM.pdf]

nature research

Corresponding author(s): Davide Marnetto, Luca Pagani
Last updated by author(s): Jan 9, 2020

Reporting Summary

Nature Research wishes to improve the reproducibility of the work that we publish. This form provides structure for consistency and transparency in reporting. For further information on Nature Research policies, see Authors & Referees and the Editorial Policy Checklist .

Statistics

For all statistical analyses, confirm that the following items are present in the figure legend, table legend, main text, or Methods section.

n/a	Confirmed	
☐	☒	The exact sample size (*n*) for each experimental group/condition, given as a discrete number and unit of measurement
☒	☐	A statement on whether measurements were taken from distinct samples or whether the same sample was measured repeatedly
☐	☒	The statistical test(s) used AND whether they are one- or two-sided<br>*Only common tests should be described solely by name; describe more complex techniques in the Methods section.*
☐	☒	A description of all covariates tested
☐	☒	A description of any assumptions or corrections, such as tests of normality and adjustment for multiple comparisons
☐	☒	A full description of the statistical parameters including central tendency (e.g. means) or other basic estimates (e.g. regression coefficient) AND variation (e.g. standard deviation) or associated estimates of uncertainty (e.g. confidence intervals)
☐	☒	For null hypothesis testing, the test statistic (e.g. *F*, *t*, *r*) with confidence intervals, effect sizes, degrees of freedom and *P* value noted<br>*Give P values as exact values whenever suitable.*
☒	☐	For Bayesian analysis, information on the choice of priors and Markov chain Monte Carlo settings
☒	☐	For hierarchical and complex designs, identification of the appropriate level for tests and full reporting of outcomes
☐	☒	Estimates of effect sizes (e.g. Cohen's *d*, Pearson's *r*), indicating how they were calculated

Our web collection on statistics for biologists contains articles on many of the points above.

Software and code

Policy information about availability of computer code

Data collection	No software was used
Data analysis	Scripts to perform the central analyses described here are stored at https://bitbucket.org/dmarnetto/ancestry-specific-partial-ps; additional software used includes: PLINK v1.90b3.27, ELAI v1.00, admix-simu (unique version), vcftools_0.1.16, R packages: lmtest_0.9-36, nonnest2_0.5-2, boot_1.3-22, PRSice_2.2.11.b

For manuscripts utilizing custom algorithms or software that are central to the research but not yet described in published literature, software must be made available to editors/reviewers. We strongly encourage code deposition in a community repository (e.g. GitHub). See the Nature Research guidelines for submitting code & software for further information.

Data

Policy information about availability of data

All manuscripts must include a data availability statement. This statement should provide the following information, where applicable:

- Accession codes, unique identifiers, or web links for publicly available datasets
- A list of figures that have associated raw data
- A description of any restrictions on data availability

The datasets analyzed during the current study are publicly available and can be accessed from the following repositories: data from 1000 Genomes Project from ftp://ftp.1000genomes.ebi.ac.uk/vol1/ftp/, data from Pagani2015 et al. at https://ega-archive.org/datasets/EGAD00001003296; data from UK Biobank at https://biobank.ndph.ox.ac.uk/showcase/ (accessed under Project \#17085); data from Estonian Biobank at https://genomics.ut.ee/en/access-biobank (accessed with Approval Number 285/T-13 obtained on 17/09/2018 by the University of Tartu Ethics Committee). GWAS summary statistics can be accessed at https://www.nealelab.is/uk-biobank and http://jenger.riken.jp/en/result for UKBB and BBJ respectively.

Field-specific reporting

Please select the one below that is the best fit for your research. If you are not sure, read the appropriate sections before making your selection.

☒ Life sciences ☐ Behavioural & social sciences ☐ Ecological, evolutionary & environmental sciences

For a reference copy of the document with all sections, see nature.com/documents/nr-reporting-summary-flat.pdf

Life sciences study design

All studies must disclose on these points even when the disclosure is negative.

Sample size	As we adopted publicly available data we most of the times included all samples available. The exception is represented by control samples from the EstBB, which were extracted from the full dataset in order to have at least the same size of the respective case cohort.<br>For UKBB we included all samples that met our requirements in a PCA/ADMIXTURE analysis We deemed our sample size sufficient as it allows to reveal polygenic score predictivity for all tested scores and phenotypes.
Data exclusions	When assessing partial polygenic scores predictivity, samples present in the training set for Type 2 Diabetes and Breast Cancer polygenic scores were removed from EstBB, and samples present in UKBB GWAS training set were removed as well from the respective testing datasets. This was pre-estabilished to avoid predictive power inflation of a Polygenic Score based on a GWAS performed on the datasets above and is a standard procedure. Other exclusions were performed due to relatedness between samples or impossibility to determine such relatedness, again as a pre-estabilished standard procedure to avoid predictability inflation. We also removed samples from UKBB dataset which had a demographic history not directly useful to the scope of this work, namely: unadmixed samples of european descent (except for a reference set); samples not showing extreme PCA coordinates; samples for which we could not define a unadmixed ancestral set as source for ELAI and as comparison (e.g. samples with partial south asian and native american descent). These were pre-estabilished criteria.
Replication	We repeated the analysis across 6 different polygenic scores for 4 different phenotypes, obtaining the same broad results in all cases. We specifically addressed result variability across approaches and traits, trying to explain possible causes in our main text.
Randomization	Control samples from the EstBB and Europeans from UKBB were randomly extracted (shuf -n bash utility) among the individuals with the information needed to fill all covariates. Incorrect local ancestry pattern were obtained through a random process described in methods.
Blinding	Blinding was not applicable to the current study.

Reporting for specific materials, systems and methods

We require information from authors about some types of materials, experimental systems and methods used in many studies. Here, indicate whether each material, system or method listed is relevant to your study. If you are not sure if a list item applies to your research, read the appropriate section before selecting a response.

Materials & experimental systems

n/a	Involved in the study	
☒	☐	Antibodies
☒	☐	Eukaryotic cell lines
☒	☐	Palaeontology
☒	☐	Animals and other organisms
☐	☒	Human research participants
☒	☐	Clinical data

Methods

n/a	Involved in the study	
☒	☐	ChIP-seq
☒	☐	Flow cytometry
☒	☐	MRI-based neuroimaging

Human research participants

Policy information about studies involving human research participants

Population characteristics	Human Research Participants data used in this study were extracted from the Estonian Biobank and UK Biobank, which are public database described elsewhere (e.g. https://www.geenivaramu.ee/en/access-biobank and https://www.ukbiobank.ac.uk/scientists-3/genetic-data/), accessible upon ethical clearance.
Recruitment	NO original recruitment was performed in this study.
Ethics oversight	Estonian Biobank data was accessed upon ethical clearance by the University of Tartu Ethics Committee, obtained on 17/09/2018 (Approval Number 285/T-13). UK Biobank data was accessed under Project #17085

Note that full information on the approval of the study protocol must also be provided in the manuscript.
